# A Nomogram of Weaning Failure for Critical Ventilated Patients in High-Altitude Areas: A Single-Center Cohort Study Using Lasso Logistic Regression

**DOI:** 10.1155/anrp/9934525

**Published:** 2025-03-11

**Authors:** Bin Wang, Li Cheng, GuoYing Lin, Ci Yang, HuiYing Zhao

**Affiliations:** ^1^Department of Critical Care Medicine, Peking University People's Hospital, Beijing, China; ^2^Department of Intensive Care Medicine, Xizang Autonomous Region People's Hospital, Lhasa, Tibet, China

**Keywords:** altitude, lasso regression, logistic model, mechanical ventilator weaning, nomograms, risk factors

## Abstract

**Objective:** This study aimed to develop a predictive model for weaning failure in critically ill patients at high altitudes.

**Methods:** Data of patients requiring invasive mechanical ventilation admitted to the Department of Intensive Care Medicine of Xizang Autonomous Region People's Hospital from January 1, 2023, to November 31, 2023, were retrospectively collected as the train set. The patients were weaned according to the conventional clinical strategy and divided into successful and failed weaning groups. Univariate analysis was performed between the weaning success and weaning failure groups. Indicators with inter-group differences were included in the Lasso regression for further screening and then included in the multivariate logistic regression analysis to establish independent risk factors. Subsequently, a nomogram prediction model was constructed. Data of patients from December 1, 2023, to April 30, 2024, were retrospectively collected as a validation set to verify the prediction model.

**Results:** A total of 226 patients were included in the train set, of which 61 (27.0%) had weaning failure. The length of intensive care unit stay, mechanical ventilation time, mortality, and medical costs of patients in the weaning failure group were higher than those in the success group. After univariate comparison and Lasso regression, hypertension, lower serum albumin, sequential organ failure assessment (SOFA) score, tidal volume, and respiratory rate were identified as independent risk factors for weaning failure. The area under the receiver operating characteristic curve was 0.895 (95% confidence interval (CI): 0.848–0.943) in the training set and 0.886 (95% CI: 0.814–0.958) in the validation set.

**Conclusions:** Hypertension, lower serum albumin, higher SOFA scores, smaller tidal volumes, and faster respiratory rates were independent risk factors for weaning failure in critically ill patients living in high-altitude areas. A prediction model for weaning failure was constructed, and it showed good prediction efficiency after verification.

**Summary**

  Quick look
  Current knowledge
• The problem of weaning ventilators has always been the focus of attention of critical care physicians.
• The partial pressure of oxygen in high-altitude areas is lower than that in plain areas, and some factors related to weaning failure encountered by patients here are different from those in plain areas.
• At present, the research on the prediction model of weaning failure in high-altitude areas is very rare.
  What this paper contributes to our knowledge
• The weaning condition of critical patients in high-altitude areas is different from that in plain areas.
• Hypertension, lower serum albumin, SOFA score, tidal volume, and respiratory rate were identified as independent risk factors for the weaning failure of these patients.
• Based on the above risk factors, the prediction model of weaning failure of critically ill patients in high-altitude areas has good prediction value.

## 1. Introduction

Mechanical ventilation (MV) is one of the most important means of life support for critically ill patients [1]. For patients undergoing invasive MV, smooth weaning largely determines their overall prognosis [2]. Therefore, intensive care doctors have been focusing on accurately evaluating patients to avoid weaning failure.

The Qinghai-Xizang Plateau has an average altitude about 4000 m, with a mainly Tibetan population [3]. The Qinghai-Xizang Plateau differs from the plain area and is characterized with low oxygen, low pressure, cold, and dry environments. People living in high-altitude areas have a greatly enhanced tolerance to low oxygen levels, and the pathophysiological mechanisms in their bodies may be different from those living in plain areas who have a relatively lower tolerance [4]. These factors can directly and indirectly affect human organs. For example, patients with acute respiratory distress syndrome in high-altitude areas have higher pulmonary capillary permeability and more severe pulmonary edema than those in plain areas, with different diagnostic criteria [5]. Therefore, there may be differences in the evaluation indicators and methods for critically ill patients in high-altitude areas when they are weaned compared with those in plain areas.

Previous studies on weaning have shown that lung disease, tidal volume, respiratory rate, consciousness, and some other factors are related to weaning failure, and some new techniques such as ultrasound have been applied to the assessment of weaning [6–8]. However there are few studies on the weaning failure of MV in high-altitude areas. The incidence and risk factors of weaning failure in critically ill patients in high-altitude regions remain unclear. Therefore, this study aimed to analyze the factors related to weaning failure in critically ill patients in high-altitude areas and build a prediction model for weaning failure to help clinicians in high-altitude areas identify patients who may have weaning failure as early as possible.

## 2. Materials and Methods

### 2.1. Study Population

Xizang Autonomous Region People's Hospital is located in Lhasa, 3650 m above sea level, and its department of intensive care medicine is the largest on the Qinghai-Xizang Plateau [9]. The intensive care unit (ICU) treats critically ill patients from various regions of Xizang, of whom 90% received invasive MV. Critically ill patients here are representative of critically ill patients in the entire Qinghai-Xizang Plateau region.

**Figure 1:**
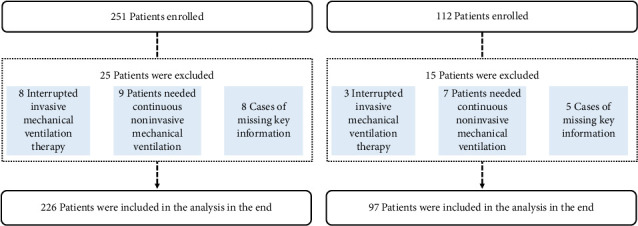
Enrollment flowchart. (a) The train set. (b) The validation set.

The train set included patients who underwent invasive MV at the Department of Intensive Care Medicine of Xizang Autonomous Region People's Hospital between January 1, 2023, and November 31, 2023. The validation set included patients from December 1, 2023, to March 31, 2024 (Figure 1). This study was approved by the Ethics Committee of Xizang Autonomous Region People's Hospital (ME-TBHP-22-34), and as a retrospective study, all patients were exempt from informed consent.

Inclusion criteria were as follows: (1) patients admitted to the Department of Intensive Care Medicine of Xizang Autonomous Region People's Hospital for invasive MV from January 1, 2023, to March 31, 2024; (2) age ≥ 18 years old; and (3) duration of invasive MV > 24 h.

The exclusion criteria were as follows: (1) patients who died before entering the weaning procedure or interrupted MV therapy for other reasons, (2) patients treated with noninvasive MV immediately after weaning, (3) patients with incomplete data, and (4) echocardiography on the first day of ICU admission indicating cardiac insufficiency.

Weaning success criteria included patients who survived weaning and did not receive invasive MV within 48 h. Weaning failure criteria included patients who required reintroduction of invasive MV or died within 48 h of weaning. Patients were divided into weaning success and weaning failure groups according to electronic medical and nursing records.

### 2.2. Variables and Definitions

Variables in this study contain lemographic characteristics, ICU assessment and history, Laboratory indicators, preweaning conditions, and prognosis variables. Demographic characteristics contains sex, age, ethnicity (Tibetan or not), body mass index, and altitude of residence. ICU assessment and history contain Acute Physiology and Chronic Health Enquiry (APACHE II) score, history of stroke, heart disease (coronary heart disease, arrhythmia, and valvular heart disease), respiratory disease (asthma, chronic obstructive pulmonary disease, and lung infection), hypertension (systolic blood pressure ≥140 mmHg and/or diastolic blood pressure ≥90 mmHg), diabetes, liver insufficiency, and smoking. Laboratory indicators were recorded within 24 h before weaning, including leukocyte platelets, hemoglobin, alanine aminotransferase, total bilirubin, serum creatinine, C-reactive protein, hypersensitive troponin I, partial pressure of carbon dioxide (PaCO2), PaO2/FiO2 (P/F) ratio, and lactic acid. Preweaning conditions contain Sequential Organ Failure Assessment (SOFA), tidal volume, respiratory rate, vasoactive drugs, and fluid balance within 24 h. Prognosis variables contain ICU stay length, MV duration, hospitalization cost, and 28-day mortality. Preweaning conditions mean the patient's general condition before weaning. Vasoactive drugs contain the infusion of norepinephrine, dopamine, and adrenaline.

### 2.3. Statistical Analysis

*R* Version 4.0.3 was used to process data. Continuous variables that did not conform to the normal distribution are represented by the median (interquartile range), and categorical variables are expressed as frequency and percentage (%). Using the “glmnet package,” univariate and Lasso logistic regression analyses were performed to verify the independent influencing factors. The nomogram model was then established using multivariate logistic regression, and the receiver operating characteristic (ROC) curve and area under the curve were used to verify the prediction efficiency of the model. The bootstrap resampling method and calibration curve were used to evaluate the consistency of the model, and the net benefit to patients was assessed using clinical decision curve analysis (DCA). The prediction model was verified using a validation set. Statistical significance was set at *p* < 0.05.

## 3. Results

A total of 251 patients were enrolled in the train set, of which 25 patients were excluded (8 patients died or were discharged during treatment, invasive MV therapy was interrupted, 9 required continuous noninvasive MV therapy after weaning, and 8 patients had incomplete data). A total of 226 patients were included in the study; among these patients, 136 patients were diagnosed with craniocerebral diseases (60.1%), 32 patients were diagnosed with abdominal diseases (14.2%), 34 patients were diagnosed with chest diseases (15%), 16 patients were diagnosed with multiple trauma (7.1%), and eight patients were diagnosed with other diseases (3.5%). The oldest age of patients included in the training set study was 85 years, the youngest was 18 years, and the average age was 49.5 years. The median age of these patients was 51 years.

Sixty one patients experienced weaning failure, and the weaning failure rate was as high as 27.0%. The length of ICU stay, MV time, and 28 day mortality of patients in the weaning failure group were higher than those in the weaning success group (*p* < 0.05) (Table 1). Although there was no significant difference in hospitalization costs between the weaning failure and weaning success groups, the median and quartiles of hospitalization costs in the weaning failure group were higher than those in the weaning success group. The weaning failure rate of critically ill patients living at high altitudes is high and has a significant adverse effect on patient prognosis.

### 3.1. Univariate Analysis

Univariate analysis showed that APACHE II score, hypertension, leukocytes, total bilirubin, C-reactive protein, hypersensitive troponin I, P/F ratio, lactic acid, SOFA score, tidal volume, respiratory rate, vasoactive drugs, and fluid balance at 24 h were statistically significant (*p* < 0.1) (Table 2).

### 3.2. Lasso Regression Analysis

**Figure 2:**
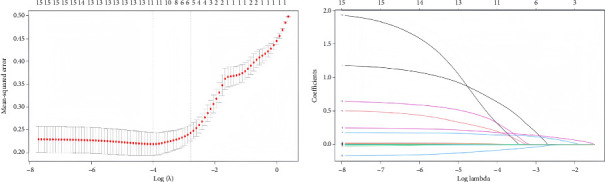
Screening for Lasso regression variable. (a) Selection process of cross-validation process parameter *λ*. (b) Dynamic process diagram of variable selection.

Indicators with statistically significant group differences in the univariate analysis were subjected to Lasso regression analysis to screen the variables. The results of the Lasso regression model showed that the optimal *λ* = 0.0613281301006316 was used to screen for five nonzero coefficient-related indices of weaning failure: hypertension, SOFA score, tidal volume, respiratory rate, and serum albumin (Figure 2).

### 3.3. Logistic Regression Analysis

The variables selected by the Lasso regression were further subjected to logistic regression analysis. The results showed that hypertension (*p*=0.025, 95% confidence interval (CI):1.143–7.070), serum albumin (*p*=0.001, 95% CI: 0.77–0.936), SOFA score (*p*=0.004, 95% CI: 1.083–1.533), tidal volume (*p* < 0.001, 95% CI: 0.986–0.995), and respiratory rate (*p* < 0.001, 95% CI: 1.169–1.415) were independent risk factors for weaning failure in critically ill patients at high altitude (Table 3).

### 3.4. Establishment and Validation of Prediction Model

**Figure 3:**
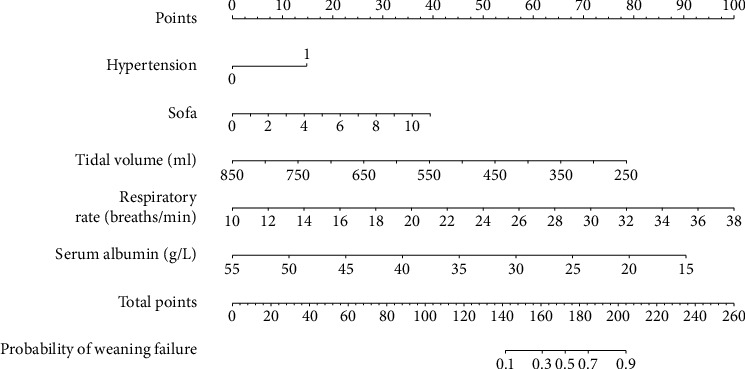
Nomogram prediction model for weaning failure of mechanical ventilation in critically ill patients at high altitude.

This study established a prediction model based on five independent risk factors: hypertension, serum albumin level, SOFA score, tidal volume, and respiratory rate (Figure 3).

A total of 112 patients were enrolled in the validation set, of which 15 were excluded (three patients died or were discharged during treatment due to interruption of invasive MV, seven patients had follow-up noninvasive ventilation after weaning, and five patients had incomplete data), and 97 patients were finally included in the validation set.

**Figure 4:**
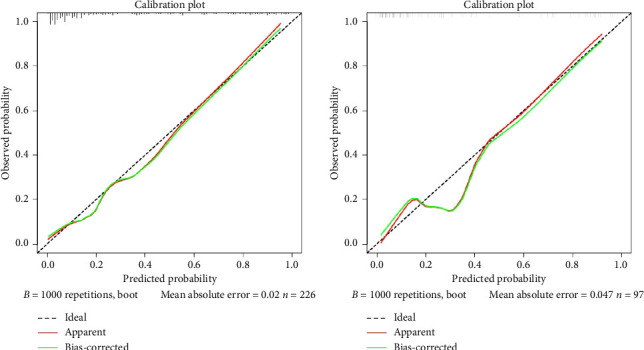
Calibration curve of the prediction model. (a) Training set. (b) Validation set.

**Figure 5:**
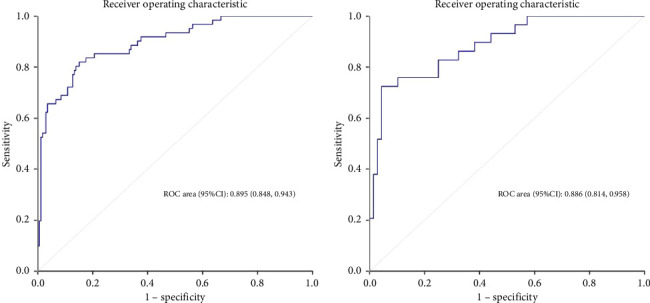
ROC curve of the prediction model. (a) Training set. (b) Validation set.

**Figure 6:**
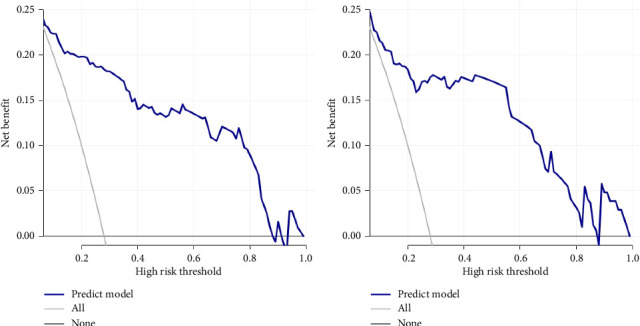
Decision curve analysis of the predictive model. (a) Training set. (b) Validation set.

Calibration curves of the prediction model for both the training and validation sets showed that the predicted values were consistent with the measured values, indicating that the model's prediction performance was good (Figure 4). The area under the ROC curve of the model training set was 0.895 (95% CI: 0.848–0.943), and the area under the ROC curve of the validation set was 0.886 (95% CI: 0.814–0.958), proving that the model had high accuracy (Figure 5). The Hosmer–Lemeshow goodness of fit was tested for the model, and the chi-square value was 5.017, *p*=0.756. The DCA shows that the clinical benefit of the model is better than that of the “All” and “None” curves (Figure 6). These results show that the model has good predictive value and clinical application. And the cutoff point of weaning failure in this nomogram prediction model is 0.3668271.

## 4. Discussion

In the present study, we established a predictive model for weaning failure in critically ill patients living in high-altitude areas. Clinicians can use this nomogram to predict the scores of the risk factors in the model and then add the scores together to obtain a total score, which can be used to obtain the corresponding risk index of weaning failure. The model was validated internally and externally and showed good prediction efficiency. We found that the risk factors for weaning failure in critically ill patients living at high altitudes included a history of hypertension, higher SOFA score, lower tidal volume, higher respiratory rate, and lower serum albumin levels. This study did not find any factors related to weaning failure specific to the plateau. The factors related to weaning failure in the plateau region are similar to those in the plain region, especially tidal volume and respiratory rate have a greater impact on weaning failure, which is also consistent with clinical experience.

MV ensures effective ventilation and optimizes oxygenation in critically ill patients. In recent years, with the progress of intensive care medicine, most critically ill patients have benefited from MV [1]. However, prolonged MV can adversely affect the patient's recovery of autonomous respiratory function and may lead to complications such as lung infections and a decline in diaphragm function. Conversely, aggressive weaning also causes weaning failure, prolongs ICU stay, and increases mortality. Therefore, there has been previous research on weaning failure [10, 11]. However, due to unique environmental factors, such as low oxygen, low pressure, and dryness, the basic physiological state and related weaning parameters of patients in high-altitude areas differ from those in plain areas [3]. At present, there are few studies on the prediction of weaning failure in critically ill patients in high-altitude regions. This study aimed to establish independent risk factors and prediction models for weaning failure at high altitudes.

Hypertension can lead to increased post-circulation load, vascular wall fiber necrosis, and atherosclerosis, reducing blood supply to organs. In addition to common heart damage, pulmonary microcirculation is also affected, leading to a high incidence of postoperative complications in hypertensive patients. Studies have shown high blood pressure is associated with lower forced expiratory volume in 1 s and forced vital capacity (FVC) [12]. A 2001 study in South Korea showed that increased systolic blood pressure was associated with decreased FVC [13]. Other studies have suggested that the relationship between blood pressure and FVC may be related to the use of hypertension medications [14]. Hypertension causes direct damage to the human respiratory and circulatory systems, resulting in hypertension patients being more prone to weaning failure.

SOFA scores describe and evaluate the degree of dysfunction or failure of individual organs in a continuous form [15]. The SOFA score represents the patient's overall organ function level, including oxygenation and consciousness, which is closely related to weaning [16]. Therefore, an increase in The SOFA score predicts the deterioration of the overall organ function of patients and also has predictive value for the occurrence of weaning failure in patients.

Tidal volume and respiratory rate are the most important ventilator regulation parameters, and their effects on weaning failure have been repeatedly demonstrated [17, 18]. Tidal volume represents the patient's ability to ventilate, and a rapid respiratory rate often indicates respiratory distress. The ratio of the respiratory rate to the tidal volume is called the rapid shallow breathing index, a classical offline indicator [19].

Many studies have confirmed that preoperative hypoalbuminemia (serum albumin concentration was lower than 35 g/L) is a risk factor for pulmonary complications [20, 21]. Some studies have shown that a decrease in albumin concentration can inhibit macrophage activation, impair the immune response, and make patients more susceptible to infection or inflammation after surgery [21]. It has also been suggested that decreased albumin concentration can reduce plasma osmotic pressure, resulting in tissue edema, interstitial fluid leakage, pulmonary congestion, and edema, and promote infection [22]. Therefore, the plasma albumin level is closely related to the respiratory function of patients, which is consistent with the results of this study: Hypoalbuminemia is an independent risk factor for weaning failure.

Yao Yan et al.'s research shows that positive end-expiratory pressure (PEEP), dynamic lung compliance, mechanical power, inspired oxygen concentration, length of ICU stay, and invasive MV duration were independent predictors of weaning failure; the predictive model they built had an area under the ROC curve of 0.828 [23]. PEEP, dynamic lung compliance, and mechanical power were not recorded in our medical records. However, their study did not include the SOFA score, an indicator that can better reflect the overall critical status of patients; therefore, the area under the ROC curve of our study was higher than that of their study. The risk factors they established included the length of ICU stay and duration of invasive MV; however, these two factors are often the result of patients' weaning failure rather than predictors. Using machine learning, Park et al. studied the predictive value of continuous ventilator parameters for weaning success during spontaneous breathing trials. The results showed that the area under the ROC curve of their model was 0.912 [24]. In their study, the model was applied by feeding the ventilator waveform and numerical data into the feature extractor. This method is novel but cannot be generalized because not all units can collect this information. This nomogram prediction model consists of five factors: hypertension, SOFA score, tidal volume, respiratory rate, and serum albumin level. The area under the ROC curve of this model reached 0.895, and after internal and external validations, the model showed good accuracy and clinical application value. At the same time, previous relevant studies were developed based on patients in plain areas. In contrast, the prediction model of this study was developed based on patients in high-altitude regions, which has certain differences. When patients in plateau areas may face the challenge of weaning failure, this model can be applied to evaluate patients to obtain the probability of weaning failure of patients and improve clinical treatment plans. And this model can be used as follows. First, determine the value corresponding to each variable, and the “Points” corresponding to this value are the scores corresponding to this variable. The downward “Probability” of the Total Points obtained by adding the scores of all the variables is the final incidence of weaning failure.

This study has some limitations. This was a retrospective study with a relatively small sample size. And in this study, the DCA curve dropped fast; considering the small sample size of the study, maybe there are some overfitting phenomena in this study. The results of this study should be verified in future large-scale prospective studies.

## 5. Conclusions

Risk factors for weaning failure in critically ill patients living at high altitudes included a history of hypertension, higher SOFA score, lower tidal volume, higher respiratory rate, and lower serum albumin levels. A predictive model for weaning failure in critically ill patients in high-altitude areas was established. The model was validated internally and showed good prediction efficiency.

## Figures and Tables

**Table 1:** Comparison of prognostic indicators.

Variables	Weaning failure group (*n* = 61)	Weaning success group (*n* = 165)	Statistical value	*p*
Length of ICU stay (days, *M* [Q_1_, Q_3_])	14 (8, 20)	9 (4, 16)	*Z* = −2.997	0.003⁣^∗^
Duration of MV (*h*, *M* [Q_1_, Q_3_])	159 (10, 308)	54 (10, 162)	*Z* = −2.446	0.014⁣^∗^
Hospitalization costs (yuan, *M* [Q_1_, Q_3_])	120,273 (72,189, 181,594)	100,910 (65,758, 161,453)	*Z* = −1.730	0.084
28 day mortality (*n* [%])	7 (11.5)	1 (0.6)	*χ* ^2^ = 12.390	< 0.001⁣^∗^

Abbreviation: ICU, intensive care unit.

⁣^∗^*p* < 0.05.

**Table 2:** Univariate analysis.

Variables	Weaning failure group (*n* = 61)	Weaning success group (*n* = 165)	Statistical value	*p*
Sex (male/female)	37/24	104/61	*χ* ^2^ = 0.107	0.744
Age (years, *M* [Q_1_, Q_3_])	53.5 (35, 61.3)	51.0 (36, 61.5)	*Z* = −0.241	0.810
Tibetan (*n* [%])	53 (86.9)	140 (84.8)	*χ* ^2^ = 0.148	0.700
BMI (kg/m2, *M* [Q_1_, Q_3_])	23.44 (20.76, 26.12)	22.86 (20.76, 25.37)	*Z* = −0.322	0.747
Altitude of residence (*m*, *M* [Q_1_, Q_3_])	3820 (3600, 4200)	3900 (3600, 4300)	*Z* = −0.317	0.751
ICU assessment and past history				
Craniocerebral diseases (*n* [%])	38 (62.3)	99 (60)	*χ* ^2^ = 0.098	0.754
APACHE II score (*M* [Q_1_, Q_3_])	16 (12, 19)	14 (10, 18)	*Z* = −2.876	0.004⁣^∗^
Stroke (*n* [%])	1 (1.6)	1 (0.6)	*Z* = 0.000	0.998
Heart disease (*n* [%])	5 (8.2)	9 (5.5)	*χ* ^2^ = 0.201	0.654
Respiratory disease (*n* [%])	1 (1.6)	7 (4.2)	*χ* ^2^ = 0.286	0.593
Hypertension (*n* [%])	32 (52.5)	53 (32.1)	*χ* ^2^ = 7.850	0.005⁣^∗^
Diabetes (*n* [%])	4 (6.6)	9 (5.5)	*χ* ^2^ = 0.100	0.752
Liver insufficiency (*n* [%])	3 (4.9)	8 (4.8)	*χ* ^2^ = 0.000	0.983
Smoking (*n* [%])	12 (19.7)	37 (22.4)	*χ* ^2^ = 0.199	0.656
Laboratory indicators				
Leukocyte (× 10^9^/L, *M* [Q_1_, Q_3_])	11.00 (8.55, 13.5)	9.50 (7.80, 13.20)	*Z* = −2.045	0.041⁣^∗^
Platelets (× 10^9^/L, *M* [Q_1_, Q_3_])	178 (99, 255)	183 (132, 274)	*Z* = −0.995	0.320
Hemoglobin (g/L, *M* [Q_1_, Q_3_])	114 (88, 148)	109 (89, 126)	*Z* = −0.779	0.436
ALT (U/L, *M* [Q_1_, Q_3_])	40.0 (20.0, 75.3)	35 (19.5, 72.3)	*Z* = −0.367	0.714
Total bilirubin (mmol/L, *M* [Q_1_, Q_3_])	20.2 (10.2, 40.0)	15.0 (9.5, 24.2)	*Z* = −1.799	0.072⁣^∗^
Creatinine (umol/L, *M* [Q_1_, Q_3_])	56.5 (33.8, 72.8)	52.0 (39.5, 68.0)	*Z* = −0.470	0.638
C-reactive protein (ng/mL, *M* [Q_1_, Q_3_])	100.0 (61.1, 137.7)	58.4 (30.8, 122.6)	*Z* = −3.033	0.002⁣^∗^
Serum albumin (g/L, *M* [Q_1_, Q_3_])	31.5 (29.6, 34.2)	33.5 (30.6, 36.8)	*Z* = −2.961	0.003⁣^∗^
Hypersensitive troponin I (ng/mL, *M* [Q_1_, Q_3_])	0.0050 (0.003, 0.0165)	0.0040 (0.0010, 0.0150)	*Z* = −1.696	0.090⁣^∗^
P/F ratio (mmhg, *M* [Q_1_, Q_3_])	224 (183, 251)	224 (218, 342)	*Z* = −4.635	< 0.001⁣^∗^
PaCO2 (mmhg, *M* [Q_1_, Q_3_])	33 (29, 37)	35 (30, 38)	*Z* = −1.395	0.163
Lactic acid (mmol/L, *M* [Q_1_, Q_3_])	1.1 (0.8, 1.5)	0.9 (0.7, 1.2)	*Z* = −1.937	0.053⁣^∗^
Preweaning conditions				
SOFA score (*M* [Q_1_, Q_3_])	4 (2, 6)	2 (0, 3)	*Z* = −6.050	< 0.001⁣^∗^
Tidal volume (ml, *M* [Q_1_, Q_3_])	390 (320, 432)	520 (455, 600)	*Z* = −7.447	< 0.001⁣^∗^
Respiratory rate (breath/min, *M* [Q_1_, Q_3_])	24 (22, 27)	18 (15, 21)	*Z* = −7.528	< 0.001⁣^∗^
Vasoactive drugs (*n* [%])	43 (70.5)	78 (47.3)	*χ* ^2^ = 9.652	0.002⁣^∗^
Fluid balance in 24 h (mL/L, *M* [Q_1_, Q_3_])	−16 (−1031, 505)	−596 (−1061, −321)	*Z* = −3.165	0.002⁣^∗^

*Note:* ALT, alanine aminotransferase, P/F ratio, PaO2/FiO2.

Abbreviations: APACHE II, Acute Physiology and Chronic Health Evaluation II; BMI, body mass index; SOFA, sequential organ failure assessment.

⁣^∗^*p* < 0.1.

**Table 3:** Multivariate logistic regression analysis.

Index	*B*	SE	*Z*	*p*	OR (95% CI)
Hypertension	1.045	0.465	5.053	0.025⁣^∗^	1.143–7.070
Serum albumin	−0.159	0.047	11.346	0.001⁣^∗^	0.777–0.936
SOFA score	0.253	0.089	8.162	0.004⁣^∗^	1.083–1.533
Tidal volume	−0.009	0.002	15.184	< 0.001⁣^∗^	0.986–0.995
Respiratory rate	0.252	0.049	26.516	< 0.001⁣^∗^	1.169–1.415

Abbreviation: SOFA, sequential organ failure assessment.

⁣^∗^*p* < 0.05.

## Data Availability

The datasets used during the current study are available from the corresponding author on reasonable request.
